# 3-Penta­none 2,4-dinitro­phenyl­hydrazone

**DOI:** 10.1107/S1600536808016887

**Published:** 2008-06-07

**Authors:** Lu-Feng Xu, Shang Shan, Wen-Long Wang, Shan-Heng Wang

**Affiliations:** a Department of Chemical Engineering, West Branch, Zhejiang University of Technology, People’s Republic of China; b College of Chemical Engineering and Materials Science, Zhejiang University of Technology, People’s Republic of China

## Abstract

Crystals of the title compound, C_11_H_14_N_4_O_4_, were obtained from a condensation reaction of 2,4-dinitro­phenyl­hydrazine and 3-penta­none. In the crystal structure, the mol­ecule, except one methyl group, displays a nearly planar structure. The imino group links to the adjacent nitro group *via* intra­molecular hydrogen bonding. The partially overlapped arrangement and face-to-face separation of 3.410 (9) Å between parallel benzene rings indicate the existence of π–π stacking between adjacent mol­ecules. The crystal structure also contains weak inter­molecular C—H⋯O hydrogen bonding.

## Related literature

For general background, see: Okabe *et al.* (1993 ▶); Shan *et al.* (2003 ▶); Shan *et al.* (2006 ▶). For related structures, see: Shan *et al.* (2008*a* ▶,*b* ▶); Cotton & Wilkinson (1972 ▶).
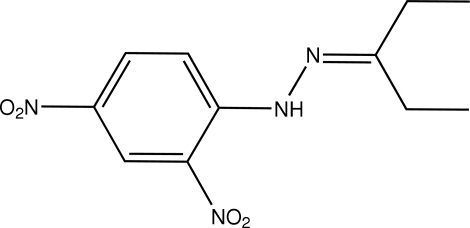


## Experimental

### 

#### Crystal data

C_11_H_14_N_4_O_4_
*M* *_r_* = 266.26
Monoclinic, 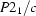

*a* = 12.5298 (15) Å
*b* = 14.089 (2) Å
*c* = 7.3983 (8) Å
β = 93.235 (12)°
*V* = 1303.9 (3) Å^3^
*Z* = 4
Mo *K*α radiation
μ = 0.10 mm^−1^
*T* = 294 (2) K
0.31 × 0.29 × 0.22 mm

#### Data collection

Rigaku R-AXIS RAPID IP diffractometer
Absorption correction: none
10943 measured reflections
2543 independent reflections
1130 reflections with *I* > 2σ(*I*)
*R*_int_ = 0.037

#### Refinement

*R*[*F* ^2^ > 2σ(*F* ^2^)] = 0.043
*wR*(*F* ^2^) = 0.136
*S* = 1.01
2543 reflections
179 parameters
H atoms treated by a mixture of independent and constrained refinement
Δρ_max_ = 0.15 e Å^−3^
Δρ_min_ = −0.12 e Å^−3^

### 

Data collection: *PROCESS-AUTO* (Rigaku, 1998 ▶); cell refinement: *PROCESS-AUTO*; data reduction: *CrystalStructure* (Rigaku/MSC, 2002 ▶); program(s) used to solve structure: *SIR92* (Altomare *et al.*, 1993 ▶); program(s) used to refine structure: *SHELXL97* (Sheldrick, 2008 ▶); molecular graphics: *ORTEP-3 for Windows* (Farrugia, 1997 ▶); software used to prepare material for publication: *WinGX* (Farrugia, 1999 ▶).

## Supplementary Material

Crystal structure: contains datablocks I, global. DOI: 10.1107/S1600536808016887/xu2428sup1.cif

Structure factors: contains datablocks I. DOI: 10.1107/S1600536808016887/xu2428Isup2.hkl

Additional supplementary materials:  crystallographic information; 3D view; checkCIF report

## Figures and Tables

**Table 1:** Hydrogen-bond geometry (Å, °)

*D*—H⋯*A*	*D*—H	H⋯*A*	*D*⋯*A*	*D*—H⋯*A*
N3—H3*N*⋯O1	0.87 (2)	1.91 (2)	2.606 (3)	136 (2)
C5—H5⋯O1^i^	0.93	2.58	3.399 (3)	147

Symmetry code: (i) 
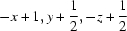
.

**Fig. 1.**
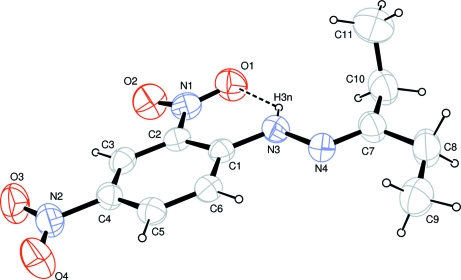
The molecular structure of the title compound with 30% probability displacement ellipsoids for non-H atoms, dashed line indicates hydrogen bonding.

**Fig. 2.**
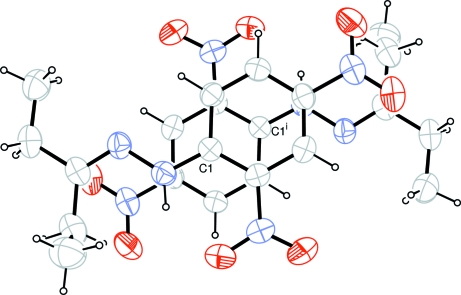
A diagram showing the partially overlapped arrangement of benzene rings [symmetry code: (i) 1 - x,1 - y,-z].

## supplementary crystallographic information

### Comment

As some phenylhydrazone derivatives have shown to be potentially DNA damaging and mutagenic agents (Okabe *et al.*, 1993), a series of new phenylhydrazone derivatives have been synthesized in our laboratory (Shan *et al.*, 2003; Shan *et al.*, 2006). As part of the ongoing investigation, the title compound has recently been prepared and its crystal structure is reported here.

The molecular structure of the title compound is shown in Fig. 1. The molecule, except the C11-methyl group, displays a nearly co-planar structure, the angle between the C11—C10 bond and C1/N3/N4/C7/C8/C9 mean plane being 61.4 (2)°. The N4—C7 bond distance is significantly shorter than N3—N4 and N3—C1 bond distances (Table 1), and indicates the typical C═N double bond. The imino group links with adjacent nitro group *via* intra-molecular hydrogen bonding (Fig. 1 and Table 2), which agrees with that found in related structures, *e.g.* furyl methyl ketone 2,4-dinitrophenylhydrazone (Shan *et al.*, 2008*a*) and 2-thiazolyl methyl ketone 2,4-dinitrophenylhydrazone (Shan *et al.*, 2008*b*).

A partially overlapped arrangement between parallel benzene rings of the adjacent molecules is illustrated in Fig. 2. The face-to-face separation of 3.410 (9) Å between C1-benzene and C1^i^-benzene rings [symmetry code: (i) 1 - *x*,1 - *y*,-*z*] is significantly shorter than van der Waals thickness of the aromatic ring (3.70 Å, Cotton & Wilkinson, 1972) and indicates the existence of π-π stacking in the crystal structure. The crystal structure also contains intermolecular weak C—H···O hydrogen bonding (Table 2).

### Experimental

2,4-Dinitrophenylhydrazine (0.4 g, 2 mmol) was dissolved in ethanol (10 ml), and H_2_SO_4_ solution (98%, 0.5 ml) was slowly added to the ethanol solution with stirring. The solution was heated at about 333 K for several min until the solution cleared. 3-Pentanone (0.17 g, 2 mmol) was then added to the above solution with continuous stirring. The mixture was refluxed for 30 min. When the solution had cooled to room temperature red powder crystals appeared. The powder crystals were separated and washed with water three times. Recrystallization from absolute ethanol solution yielded well shaped single crystals of the title compound.

### Refinement

Imino H atom was located in a difference Fourier map and refined isotropically. Methyl H atoms were placed in calculated positions with C—H = 0.96 Å and torsion angles were refined to fit the electron density, *U*_iso_(H) = 1.5*U*_eq_(C). Other H atoms were placed in calculated positions with C—H = 0.93 (aromatic) and 0.97 Å (methylene), and refined in riding mode with *U*_iso_(H) = 1.2*U*_eq_(C).

### Crystal data

**Table:**

C_11_H_14_N_4_O_4_	*F*_000_ = 560
*M**_r_* = 266.26	*D*_x_ = 1.356 Mg m^−^^3^
Monoclinic, *P*2_1_/*c*	Mo *K*α radiation λ = 0.71069 Å
Hall symbol: -P 2ybc	Cell parameters from 2556 reflections
*a* = 12.5298 (15) Å	θ = 3.5–25.0º
*b* = 14.089 (2) Å	µ = 0.11 mm^−^^1^
*c* = 7.3983 (8) Å	*T* = 294 (2) K
β = 93.235 (12)º	Prism, red
*V* = 1303.9 (3) Å^3^	0.31 × 0.29 × 0.22 mm
*Z* = 4	

### Data collection

**Table:**

Rigaku R-AXIS RAPID IP diffractometer	2543 independent reflections
Radiation source: fine-focus sealed tube	1130 reflections with *I* > 2σ(*I*)
Monochromator: graphite	*R*_int_ = 0.037
Detector resolution: 10.00 pixels mm^-1^	θ_max_ = 26.0º
*T* = 294(2) K	θ_min_ = 3.1º
ω scans	*h* = −15→15
Absorption correction: none	*k* = −17→17
10943 measured reflections	*l* = −9→8

### Refinement

**Table:**

Refinement on *F*^2^	Hydrogen site location: inferred from neighbouring sites
Least-squares matrix: full	H atoms treated by a mixture of independent and constrained refinement
*R*[*F*^2^ > 2σ(*F*^2^)] = 0.043	*w* = 1/[σ^2^(*F*_o_^2^) + (0.0627*P*)^2^ + 0.0303*P*] where *P* = (*F*_o_^2^ + 2*F*_c_^2^)/3
*wR*(*F*^2^) = 0.136	(Δ/σ)_max_ < 0.001
*S* = 1.01	Δρ_max_ = 0.15 e Å^−^^3^
2543 reflections	Δρ_min_ = −0.12 e Å^−^^3^
179 parameters	Extinction correction: SHELXL, Fc^*^=kFc[1+0.001xFc^2^λ^3^/sin(2θ)]^-1/4^
Primary atom site location: structure-invariant direct methods	Extinction coefficient: 0.014 (2)
Secondary atom site location: difference Fourier map	

### Special details

**Table:**

Geometry. All e.s.d.'s (except the e.s.d. in the dihedral angle between two l.s. planes) are estimated using the full covariance matrix. The cell e.s.d.'s are taken into account individually in the estimation of e.s.d.'s in distances, angles and torsion angles; correlations between e.s.d.'s in cell parameters are only used when they are defined by crystal symmetry. An approximate (isotropic) treatment of cell e.s.d.'s is used for estimating e.s.d.'s involving l.s. planes.
Refinement. Refinement of *F*^2^ against ALL reflections. The weighted *R*-factor *wR* and goodness of fit *S* are based on *F*^2^, conventional *R*-factors *R* are based on *F*, with *F* set to zero for negative *F*^2^. The threshold expression of *F*^2^ > σ(*F*^2^) is used only for calculating *R*-factors(gt) *etc*. and is not relevant to the choice of reflections for refinement. *R*-factors based on *F*^2^ are statistically about twice as large as those based on *F*, and *R*- factors based on ALL data will be even larger.

### Fractional atomic coordinates and isotropic or equivalent isotropic displacement parameters (Å^2^)

**Table:**

	*x*	*y*	*z*	*U*_iso_*/*U*_eq_	
N1	0.49005 (19)	0.29067 (16)	0.0607 (3)	0.0817 (6)	
N2	0.79233 (17)	0.4911 (2)	0.2228 (3)	0.0928 (7)	
N3	0.35067 (15)	0.43228 (17)	0.2123 (3)	0.0742 (6)	
N4	0.28589 (14)	0.50533 (14)	0.2635 (2)	0.0758 (6)	
O1	0.39384 (16)	0.27125 (13)	0.0591 (3)	0.1056 (6)	
O2	0.55348 (16)	0.23773 (14)	−0.0069 (3)	0.1145 (7)	
O3	0.84951 (15)	0.42733 (18)	0.1731 (3)	0.1230 (8)	
O4	0.82618 (14)	0.56837 (18)	0.2750 (3)	0.1248 (8)	
C1	0.45801 (16)	0.44522 (16)	0.2116 (2)	0.0610 (6)	
C2	0.52770 (16)	0.37825 (16)	0.1427 (2)	0.0636 (6)	
C3	0.63704 (17)	0.39319 (17)	0.1474 (3)	0.0707 (6)	
H3	0.6823	0.3478	0.1019	0.085*	
C4	0.67726 (16)	0.47555 (18)	0.2196 (3)	0.0687 (6)	
C5	0.61123 (17)	0.54410 (17)	0.2870 (3)	0.0696 (6)	
H5	0.6402	0.6000	0.3352	0.083*	
C6	0.50387 (17)	0.52968 (16)	0.2827 (3)	0.0665 (6)	
H6	0.4599	0.5763	0.3275	0.080*	
C7	0.18529 (19)	0.4892 (2)	0.2520 (3)	0.0854 (7)	
C8	0.11384 (19)	0.5688 (2)	0.3035 (4)	0.1065 (9)	
H8A	0.0615	0.5796	0.2040	0.128*	
H8B	0.0752	0.5485	0.4068	0.128*	
C9	0.1670 (2)	0.6608 (2)	0.3497 (4)	0.1228 (11)	
H9A	0.2204	0.6513	0.4462	0.184*	
H9B	0.1148	0.7054	0.3874	0.184*	
H9C	0.2001	0.6851	0.2453	0.184*	
C10	0.1317 (2)	0.3981 (2)	0.1833 (4)	0.1104 (10)	
H10A	0.0593	0.4123	0.1380	0.132*	
H10B	0.1703	0.3733	0.0836	0.132*	
C11	0.1285 (3)	0.3250 (3)	0.3265 (4)	0.1350 (11)	
H11A	0.2002	0.3082	0.3670	0.202*	
H11B	0.0920	0.2697	0.2788	0.202*	
H11C	0.0914	0.3496	0.4264	0.202*	
H3N	0.3286 (18)	0.3785 (18)	0.167 (3)	0.092 (9)*	

### Atomic displacement parameters (Å^2^)

**Table:**

	*U*^11^	*U*^22^	*U*^33^	*U*^12^	*U*^13^	*U*^23^
N1	0.0977 (15)	0.0693 (15)	0.0780 (13)	0.0007 (13)	0.0042 (12)	−0.0036 (11)
N2	0.0699 (15)	0.116 (2)	0.0930 (15)	−0.0022 (14)	0.0047 (11)	0.0069 (14)
N3	0.0693 (13)	0.0696 (15)	0.0838 (13)	−0.0045 (12)	0.0044 (10)	0.0017 (11)
N4	0.0664 (12)	0.0779 (15)	0.0833 (12)	0.0052 (10)	0.0063 (9)	0.0082 (10)
O1	0.1008 (13)	0.0832 (14)	0.1323 (15)	−0.0184 (11)	0.0006 (11)	−0.0159 (11)
O2	0.1273 (15)	0.0911 (15)	0.1265 (16)	0.0073 (12)	0.0198 (12)	−0.0373 (12)
O3	0.0742 (11)	0.143 (2)	0.1533 (19)	0.0159 (12)	0.0156 (11)	−0.0054 (14)
O4	0.0865 (13)	0.138 (2)	0.1503 (18)	−0.0311 (13)	0.0091 (11)	−0.0206 (15)
C1	0.0659 (13)	0.0637 (16)	0.0532 (11)	−0.0012 (11)	0.0020 (10)	0.0093 (10)
C2	0.0733 (14)	0.0604 (15)	0.0570 (12)	0.0004 (12)	0.0022 (10)	0.0053 (11)
C3	0.0761 (15)	0.0749 (17)	0.0613 (13)	0.0131 (12)	0.0072 (11)	0.0077 (11)
C4	0.0618 (13)	0.0807 (17)	0.0637 (13)	−0.0006 (13)	0.0040 (10)	0.0075 (12)
C5	0.0746 (15)	0.0700 (16)	0.0637 (12)	−0.0068 (12)	0.0010 (11)	0.0018 (11)
C6	0.0700 (14)	0.0647 (15)	0.0647 (13)	0.0042 (11)	0.0043 (10)	0.0014 (11)
C7	0.0660 (15)	0.099 (2)	0.0907 (17)	−0.0043 (14)	0.0038 (12)	0.0060 (14)
C8	0.0719 (16)	0.124 (3)	0.124 (2)	0.0170 (17)	0.0121 (15)	0.0007 (19)
C9	0.104 (2)	0.103 (3)	0.163 (3)	0.0176 (18)	0.0196 (19)	−0.003 (2)
C10	0.0770 (17)	0.136 (3)	0.118 (2)	−0.0064 (17)	0.0036 (16)	0.002 (2)
C11	0.147 (3)	0.123 (3)	0.135 (3)	−0.027 (2)	0.015 (2)	−0.001 (2)

### Geometric parameters (Å, °)

**Table:**

N1—O2	1.218 (2)	C5—H5	0.9300
N1—O1	1.235 (2)	C6—H6	0.9300
N1—C2	1.442 (3)	C7—C8	1.497 (4)
N2—O3	1.219 (3)	C7—C10	1.523 (4)
N2—O4	1.222 (3)	C8—C9	1.489 (4)
N2—C4	1.457 (3)	C8—H8A	0.9700
N3—C1	1.358 (3)	C8—H8B	0.9700
N3—N4	1.377 (3)	C9—H9A	0.9600
N3—H3N	0.87 (2)	C9—H9B	0.9600
N4—C7	1.279 (3)	C9—H9C	0.9600
C1—C2	1.401 (3)	C10—C11	1.479 (4)
C1—C6	1.410 (3)	C10—H10A	0.9700
C2—C3	1.385 (3)	C10—H10B	0.9700
C3—C4	1.362 (3)	C11—H11A	0.9600
C3—H3	0.9300	C11—H11B	0.9600
C4—C5	1.383 (3)	C11—H11C	0.9600
C5—C6	1.359 (3)		
			
O2—N1—O1	121.3 (2)	N4—C7—C8	116.9 (2)
O2—N1—C2	119.4 (2)	N4—C7—C10	125.9 (2)
O1—N1—C2	119.3 (2)	C8—C7—C10	117.2 (2)
O3—N2—O4	123.5 (2)	C9—C8—C7	116.4 (2)
O3—N2—C4	118.8 (3)	C9—C8—H8A	108.2
O4—N2—C4	117.7 (3)	C7—C8—H8A	108.2
C1—N3—N4	120.0 (2)	C9—C8—H8B	108.2
C1—N3—H3N	114.2 (16)	C7—C8—H8B	108.2
N4—N3—H3N	125.4 (16)	H8A—C8—H8B	107.4
C7—N4—N3	116.3 (2)	C8—C9—H9A	109.5
N3—C1—C2	123.3 (2)	C8—C9—H9B	109.5
N3—C1—C6	119.7 (2)	H9A—C9—H9B	109.5
C2—C1—C6	117.06 (19)	C8—C9—H9C	109.5
C3—C2—C1	121.6 (2)	H9A—C9—H9C	109.5
C3—C2—N1	116.1 (2)	H9B—C9—H9C	109.5
C1—C2—N1	122.2 (2)	C11—C10—C7	112.2 (2)
C4—C3—C2	118.9 (2)	C11—C10—H10A	109.2
C4—C3—H3	120.6	C7—C10—H10A	109.2
C2—C3—H3	120.6	C11—C10—H10B	109.2
C3—C4—C5	121.4 (2)	C7—C10—H10B	109.2
C3—C4—N2	118.6 (2)	H10A—C10—H10B	107.9
C5—C4—N2	120.0 (2)	C10—C11—H11A	109.5
C6—C5—C4	119.9 (2)	C10—C11—H11B	109.5
C6—C5—H5	120.0	H11A—C11—H11B	109.5
C4—C5—H5	120.0	C10—C11—H11C	109.5
C5—C6—C1	121.1 (2)	H11A—C11—H11C	109.5
C5—C6—H6	119.4	H11B—C11—H11C	109.5
C1—C6—H6	119.4		

### Hydrogen-bond geometry (Å, °)

**Table:**

*D*—H···*A*	*D*—H	H···*A*	*D*···*A*	*D*—H···*A*
N3—H3N···O1	0.87 (2)	1.91 (2)	2.606 (3)	136 (2)
C5—H5···O1^i^	0.93	2.58	3.399 (3)	147

Symmetry codes: (i) −*x*+1, *y*+1/2, −*z*+1/2.
